# Impact of FAPI-46/dual-tracer PET/CT imaging on radiotherapeutic management in esophageal cancer

**DOI:** 10.1186/s13014-024-02430-9

**Published:** 2024-04-04

**Authors:** Simone Wegen, Karina Claus, Philipp Linde, Johannes Rosenbrock, Maike Trommer, Thomas Zander, Armin Tuchscherer, Christiane Bruns, Hans Anton Schlößer, Wolfgang Schröder, Marie-Lisa Eich, Thomas Fischer, Klaus Schomäcker, Alexander Drzezga, Carsten Kobe, Katrin Sabine Roth, Jasmin Josefine Weindler

**Affiliations:** 1 Department of Radiation Oncology, Cyberknife and Radiotherapy, Faculty of Medicine, University Hospital Cologne, Cologne, Germany; 2 Center for Integrated Oncology (CIO), Faculty of Medicine and University Hospital, University of Cologne, Cologne, Germany; 3 Department I of Internal Medicine, Faculty of Medicine and University Hospital, University of Cologne, Cologne, Germany; 4 Department of General, Visceral, Cancer and Transplantation Surgery, Faculty of Medicine, with University Hospital Cologne, Cologne, Germany; 5 Institute of Pathology, Faculty of Medicine, University Hospital Cologne, Cologne, Germany; 6 Department of Nuclear Medicine, Faculty of Medicine and University Hospital Cologne, University of Cologne, Cologne, Germany; 7 Institute of Neuroscience and Medicine, Molecular Organization of the Brain, Forschungszentrum Jülich, INM-2, Cologne, Germany; 8 Department of Radiation Oncology, Olivia Newton-John Cancer Wellness & Research Centre, Austin Health, Melbourne, Australia; 9 Center for Molecular Medicine Cologne, Faculty of Medicine and University Hospital Cologne, University of Cologne, Cologne, Germany

**Keywords:** FDG, Esophageal cancer, FAPI-46, Radiotherapy planning, PET-based

## Abstract

**Background:**

Fibroblast activation protein (FAP) is expressed in the tumor microenvironment (TME) of various cancers. In our analysis, we describe the impact of dual-tracer imaging with Gallium-68-radiolabeled inhibitors of FAP (FAPI-46-PET/CT) and fluorodeoxy-D-glucose (FDG-PET/CT) on the radiotherapeutic management of primary esophageal cancer (EC).

**Methods:**

32 patients with EC, who are scheduled for chemoradiation, received FDG and FAPI-46 PET/CT on the same day (dual-tracer protocol, 71%) or on two separate days (29%) We compared functional tumor volumes (FTVs), gross tumor volumes (GTVs) and tumor stages before and after PET-imaging. Changes in treatment were categorized as “minor” (adaption of radiation field) or “major” (change of treatment regimen). Immunohistochemistry (IHC) staining for FAP was performed in all patients with available tissue.

**Results:**

Primary tumor was detected in all FAPI-46/dual-tracer scans and in 30/32 (93%) of FDG scans. Compared to the initial staging CT scan, 12/32 patients (38%) were upstaged in nodal status after the combination of FDG and FAPI-46 PET scans. Two lymph node metastases were only visible in FAPI-46/dual-tracer. New distant metastasis was observed in 2/32 (6%) patients following FAPI-4 -PET/CT. Our findings led to larger RT fields (“minor change”) in 5/32 patients (16%) and changed treatment regimen (“major change”) in 3/32 patients after FAPI-46/dual-tracer PET/CT. GTVs were larger in FAPI-46/dual-tracer scans compared to FDG-PET/CT (mean 99.0 vs. 80.3 ml, respectively (*p* < 0.001)) with similar results for nuclear medical FTVs. IHC revealed heterogenous FAP-expression in all specimens (mean H-score: 36.3 (SD 24.6)) without correlation between FAP expression in IHC and FAPI tracer uptake in PET/CT.

**Conclusion:**

We report first data on the use of PET with FAPI-46 for patients with EC, who are scheduled to receive RT. Tumor uptake was high and not depending on FAP expression in TME. Further, FAPI-46/dual-tracer PET had relevant impact on management in this setting. Our data calls for prospective evaluation of FAPI-46/dual-tracer PET to improve clinical outcomes of EC.

## Introduction

Esophageal carcinomas (EC) account for approximately 1% of all malignant diseases and 2% of all cancer-related deaths in Germany [1] and the incidence of adenocarcinoma is growing. Exact tumor staging is crucial to select the right treatment option for each patient. 30–40% of patients are in a resectable tumor stage when first diagnosed. Both, undertreatment of patients with undetected metastatic lesions (cM1) as well as overtreatment (intense chemotherapy regimens) in patients with early-stage EC and wrong positive findings in conventional staging can impact patients’ prognosis and outcomes.

FDG PET/CT is a helpful tool for tumor delineation in radiation oncology (RO) and has become, if available, a recommended diagnostic tool in many countries [2], yet there is no consensus on radiotherapeutic management of FDG-positive lesions. Sensitivity for detection of lymph node (LN) metastasis in FDG PET/CT is described as 49%, specificity 87%% and accuracy 68% [3]. In comparison, endobronchial ultrasound (EUS) and fine-needle aspiration of LN show higher rates of pooled specificity and accuracy (81% and 77%, respectively). Given poor prognosis of EC, there is great interest in identifying novel diagnostic targets. In several tumor entities, FAPI-labelled PET tracers showed to be superior to conventional FGD PET/CT [4]. There is first evidence, that initial tumor staging with FAPI PET/CT shows good diagnostic performance in detecting primary tumor and lymph node metastases in patients with EC [5]. 

In a newly developed dual-tracer protocol, we showed greater Gross Tumor Volumes (GTVs) derived from FAPI-46/dual-tracer PET/CT in a case series of cervical, head and neck and esophageal cancer patients [6, 7] leading to larger irradiation fields. Our aim in this analysis is to describe the impact of imaging with radiolabeled inhibitors of FAPI ([^68^Ga]Ga-FAPI-46-PET/CT) and fluorodeoxy-D-glucose ([^18^F]F-FDG-PET/CT) on radiotherapeutic management of esophageal cancer (EC).

## Patients and methods

### Patient cohort, dual-tracer protocol and PET/CT imaging

Our retrospective analysis included 32 patients with primary esophageal cancer who received PET/CT scans between May 2020 and May 2023 (for patient characteristics, see Table 1). Of these, 72% (23 patients) underwent single session/dual-tracer PET/CT protocol (both scans on the same day) while 9 patients received their FDG- and FAPI-46 PET/CTs on two different time points. All scans were performed for cancer staging prior to radiotherapy. Dual-tracer PET/CT imaging was carried out according to a previously published protocol [6] and it allows administering both PET tracers (FAPI-46 and FDG) in one appointment.

**Table 1:** Patient characteristics

	Overall (*N* = 32)
**Age**	
Mean (SD)	64.6 (12.3)
**Sex**	
female	6 (18.8%)
male	26 (81.3%)
**BMI**	
Mean (SD)	24.2 (5.38)
**Smoker**	
current	14 (43.8%)
former	11 (34.4%)
never	7 (21.9%)
**Tumor grade**	
1	3 (9.4%)
2	19 (59.4%)
3	9 (28.1%)
missing	1 (3.1%)
**Histology**	
adenocarcinoma	15 (46.9%)
SCC	17 (53.1%)
**Intended Treatment** (pre-PET)	
definitive CRT	15 (46.9%)
neoadjuvant CRT	16 (50.0%)
other	1 (3.1%)
**N-stage** (pre-PET)	
0	9 (28.1%)
1	10 (31.3%)
2	6 (18.8%)
3	7 (21.9%)
**M-stage** (pre-PET**)**	
0	30 (93.8%)
1*	2 (6.3%)

SD = standard deviation, BMI = body mass index, SCC = squamous cell carcinoma, CRT = chemoradiotherapy, PET = Positron Emission Tomography *= distal EC with suspicion of a cervical (supraclavicular) metastasis

The acquisition of the PET/CT was in a supine position in craniocaudal direction, whole body, starting at the skull base until mid-thigh. The mean time interval between application of FDG and scanning was 64.8 ± 14.2 min. In case of dual-tracer PET/CT protocol, after completion of FDG-PET/CT scan with 234.9 ± 59.9 MBq (mean), we injected 182.3 ± 43.1 MBq FAPI-46 (mean). The subsequent dual-tracer PET/CT was performed 18 ± 22 min after injection. When FAPI-46 PET/CT was performed on a different day as the FDG-PET/CT, patients received 166.2 ± 36.4 MBq FAPI-46 and FAPI-46 PET/CT was performed 44.2 ± 22.6 min later (see Table 2). For improved subsequent radiotherapy planning and matching of the images, patients wore immobilization devices for head, arms, back and knees to ensure a good correlation between PET/CT and radiotherapy. Patients with high (cervical) esophageal cancer received a thermoplastic mask. PET/CT scans were evaluated by two independent reviewers (specialists for nuclear medicine). A correlation with the CT was performed to exclude unspecific findings.

**Table 2:** Scan data

Patients (*n* = 32)	Mean ± SD	Number			
Malignant findings in PET/CT		FDG		FAPI-46	
		CR < 4.0	CR > 4.0	CR < 4.0	CR > 4.0
Tumors (*n* = 32)		2	30	2	30
Metastasis (*n* = 25)		7	18	1	24
Lymph node metastasis		6	11	1	16
Liver metastasis		1	1	0	2
Gastric metastasis / second tumor		0	3	0	3
Bone metastasis		0	1	0	1
Adrenal gland metastasis		0	1	0	1
Muscle metastasis		0	1	0	1
Time between injection FAPI and Scan (min)	21.8 ± 22.4				
Time between injection FDG and Scan (min)	64.8 ± 14.2				

Injection data of FAPI-46/FDG and count of malignant findings. FTV measurement was performed by using a fixed threshold of CR 4.0 or in case of dual-tracer PET/CT a CR-adapted threshold as described before. Here, we show an overview of all lesions with a CR > 4.0 that are thus measurable for FTV. SD = standard deviation, CR = count rate, FDG = fluorodeoxy-D-glucose, FAPI = fibroblast activation protein inhibitor

### Count rates (CR) and functional tumor volume (FTV) measurements

Volumes of interest (VOIs) were drawn around the primary tumor and, if present, the metastases, to determine the maximum count rates (CRmax) and/or peak count rates (CRpeak) of the lesions. Mean count rates (CRmean) of reference tissue (mediastinal blood pool and liver) were measured with VOIs of 1 cm diameter in the descending thoracic aorta (representing mediastinal blood pool) and VOIs of 2 cm diameter in the right liver lobe.

FTVs of tumors and metastasis were measured in the single- and dual-tracer PET/CT scans. FTVs were calculated in all PET scans using the SyngoVia workstation (Siemens, Erlangen, Germany), taking a fixed threshold of CR of 4.0 (FTV4.0) or a CR-adapted threshold (defindes as the 2.5-fold of CRmean in the bloodpool (BP) (= FTVBPadapted). The latter was necessary to compensate for increased CR values sometimes occurring in the reference and background tissue in patients with dual-tracer PET/CT. FTV-measurements were performed in the primary tumor (FTVtumor), in nodal/distant metastasis (FTVmetastasis) and, if present, in secondary tumors (FTV2nd_tumor, *n* = 3).

All clinical investigations were conducted according to Declaration of Helsinki principles. All procedures were performed in compliance with the regulations of the responsible local authorities and the local institutional review board waived the requirement for additional approval owing to the retrospective character of this study (23-1254-retro). All patients gave written informed consent to PET imaging.

Descriptive statistics were used to present patient characteristics and results. For statistical analysis, a Wilcoxon matched-pairs signed-rank test was performed to detect significant differences between continuous variables. A p-value of less than 0.05 (*p* < 0.05) was considered significant. To measure the strength of the correlation, Pearson’s correlation coefficient was used. All statistical analyses were performed using SPSS-Statistics v.28.0.1.1 (IBM, Armonk, NY, USA).

### Target volume delineation

Varian medical systems (Siemens Healthineers, Erlangen, Germany) treatment planning system (TPS) ‘Eclipse’ was used for target volume delineation and matching of the present imaging. By ensuring that the positioning for scanning of all patients was the same as that used for subsequent radiotherapy, we were able to achieve exact matching of the images. Radiotherapy reference points were marked on the patients’ skin.

For comparison of target volume delineations, two GTVs were created independently. First, a GTV based on FDG PET/CT was created and second, a GTV based on either FAPI-46 PET/CT alone or on base of the combination of both scans (FAPI-46/dual-tracer PET/CT) was set up. For PET/CT based contouring, a window of SUVmax 0–5 was employed. GTVs for oesophageal cancer were contoured according to the practical guideline of the American expert consensus group for contouring of esophageal and gastroesophageal junction cancer [8] or according to the European target volume delineation atlas for the neoadjuvant radiation treatment [9] and approved by a group of board-certified radiation oncologists.

Potential pitfalls or known benign sites of FAPI-uptake were taken into consideration when interpreting discrepant tumor areas (dual-tracer or FAPI-46 **+** but FDG**-**). Moreover, lesions were correlated with CT-imaging in favour to confirm possible pathological findings of PET/CT scans and to safely ensure pathological interpretation of PET-positive lesions. Upstaging or treatment decisions were based on the collected information gained from all used diagnostic methods. Indeed, all such decisions were discussed in multidisciplinary meetings with medical oncologists, gastroenterologists, surgeons, physicians for nuclear medicine and radiation oncologists in consensus by taking the results of clinical examination, EUS, PET/CT imaging and CT imaging into consideration.

### Tumor staging and change of treatment after PET

In esophageal cancer, endobronchial ultrasound examination (EUS) is the accepted standard to assess T-stage (depth of invasion), so it was not determined in the PET-scans. For N-staging, we first defined the pre-PET (CT-based) N-stage, then counted PET-positive lymph nodes in both FDG PET/CT and FAPI-46/dual-tracer PET/CT and classified them as following: 1–2 positive regional lymph nodes: cN1, 3–6 positive regional lymph nodes: cN2, > 7 positive regional lymph nodes: cN3, according to the 8th edition AJCC/UICC TNM-staging for esophageal cancer [10]. In M-staging, we defined a pretherapeutic M-stage and compared it to our findings in the PET scans (cM0: no distant metastasis, cM1a: distant lymph nodes (cervical/coeliac), cM1b all other distant metastasis) [10]. Findings with a potential impact on therapy regimen were discussed in the interdisciplinary tumor board meetings. Patients with lesions that remained unclear (e.g. one soft tissue metastasis in a patient’s gluteal muscle) had to receive a biopsy of the suspicious lesion. To better understand the impact of FAPI-46/dual-tracer PET/CT in radiotherapy (RT) planning, we categorized our findings into “minor change” meaning any change (usually enlargement) of RT-field after PET due to more lymph nodes or different lymph node regions that need to be covered by the RT field and “major changes”, meaning every change of treatment regimen (e.g. from neoadjuvant to definitive chemoradiation or from curative to palliative treatment).

### Immunohistochemistry

From our cohort, biopsy specimens of the primary esophageal tumor (squamous cell carcinoma (SCC) and adenocarcinoma (AC)) were selected for further analysis. Whole slides were automatically stained with the Leica BOND-MAX (Leica Biosystems, Wetzlar, Germany) for FAP (Abcam, clone EPR20021) as previously described and whole slide images were digitized with the with the Aperio GT 450 DX (Leica Biosystems, Wetzlar, Germany). Analysis was carried out in QuPath v0.4.3 as previously described [11]. Tumor and tumor stromal areas were annotated. Positive cell detection was performed under the following settings: setup parameters: detection image optical density sum, requested pixel size 1 μm; nucleus parameters: background radius 8 μm, median filter radius 0 μm, sigma 2 μm, minimum area 12 µm2, maximum area 400 µm2; intensity parameters: threshold 0.1, max background intensity 2; and cell parameters: cell expansion 5 μm, cell nucleus included. Number of positive cells and H-Scores were calculated in QuPath for all available cases.

## Results

### Patient cohort

**Fig. 1:**
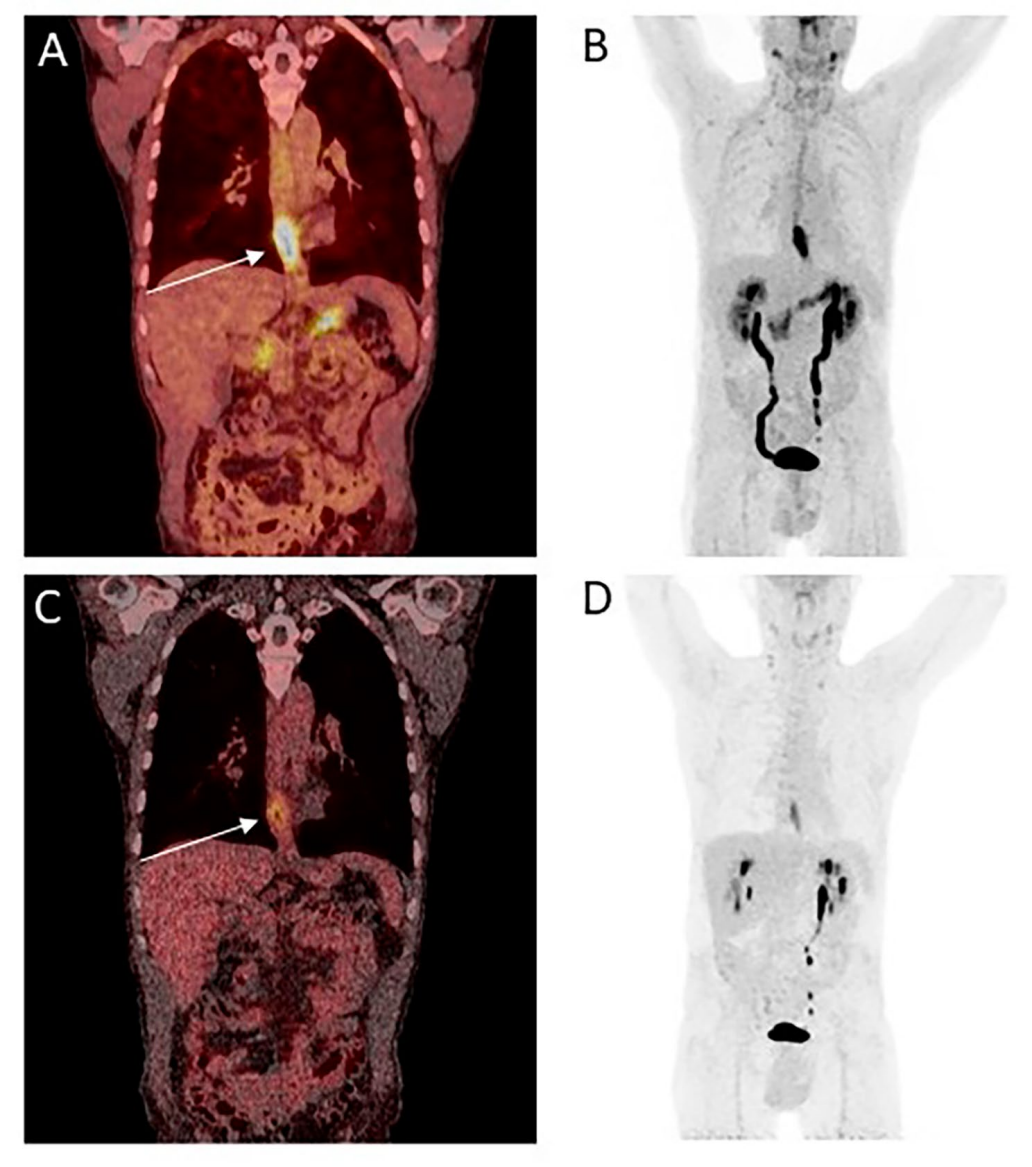
Patient with newly diagnosed distal esophageal cancer 49-year-old patient with newly diagnosed distal esophageal cancer (adenocarcinoma, G2). The primary tumor showed strong tracer uptake in the FAPI-46/dual-tracer PET/CT (**A** = coronal fusion, **B** = Maximum Intensity Projection (MIP)), whereas it shows smaller tracer uptake in FDG PET/CT (**C** = coronal fusion, same layer as A, **D** = MIP). White arrow indicating tumor. There was no sight of nodal or distant metastasis

For this study, PET/CTs of 25 male and 7 female patients between 36 and 84 years (mean 64.6 ± 12.3 years) were evaluated. Patients had histologically confirmed EC prior to imaging and were referred by the local multidisciplinary tumor board for neoadjuvant or definitive chemoradiation. Majority of patients had grade 2 (G2) tumors (59.4%) and were active (43.8%) or former (34.4%) smokers. Histopathologically, the distribution was about the same between SCC (53.1%) and adenocarcinoma (46.9%). For further patient characteristics and an example of a patient with distal EC who underwent both FDG and FAPI-46/dual-tracer PET/CT, see Table 1; Fig. 1.

All 32 patients received FDG PET/CT and FAPI-46 PET/CT prior to radiotherapy. Nine out of 32 patients (28%) received the scans on two different days, but, due to improvement of our protocol, after the first 7 patients we decided to continue PET staging according to the dual-tracer protocol and only performed two separate scans, when we faced logistical difficulties in two patients afterwards. PET/CT examinations were performed without any records of side effects.

To define the impact of FAPI-46 PET/CT in clinical use, we decided to analyze the PET scans from a nuclear medicine (biodistribution), radiation oncology and histopathological perspective.

### PET/CT scan parameters and lesion detection in both scans

All patients had biopsy-proven esophageal cancer with a location in the gastroesophageal junction in 37,5% (12/32). Three patients (9,3%) were suspected to have an additional tumor in the stomach. In one case, it was not possible to distinguish between a (large) lymph node metastasis in proximity to the stomach and a gastric tumor (secondary localisation).

All primary esophageal tumors (*n* = 32, 100%) could be identified in FAPI-46-PET/CT (both single and dual-tracer). In all patients investigated, we were able to detect 24 metastases (lymph node: 17, liver: 2, gastric: 3, bone: 1, adrenal: 1, see Table 2). Two mediastinal lymph node metastases were only visible in FAPI-46 (dual-tracer), not in FDG. In contrast, one lymph node metastasis with projection to the supraclavicular region has shown FDG-uptake with no tracer accumulation in the FAPI-46 PET/CT.

In some patients, we observed unspecific tracer accumulation in FAPI-46 PET/CT around joints (e.g. hip and shoulder), intramuscularly and in tendons. These findings were carefully checked and could be evaluated as benign findings (degenerative joint changes, a bursitis or tendinopathy). In one patient, FAPI-46 PET/CT showed a strong liver enhancement and hepatomegaly. Based on the patient’s history of alcohol consumption and in correlation with elevated liver enzymes, we assumed the presence of liver cirrhosis and arranged for further investigation (ultrasound). Liver cirrhosis was confirmed.

### Functional tumor volumes (FTVs) in FDG PET/CT vs. FAPI-46 PET/CT (single/dual-tracer)

Automated FTV measurements and BP-adjusted FTV measurements were possible for all PET/CT scans (FDG and FAPI-46). In two patients, primary tumors could not be detected in the FDG scans with SyngoVia Lesion Scout due to lack of tracer accumulation. A total of seven metastases (visible lesions on FDG-PET/CT) could not be measured by the SyngoVia Lesion Scout (vs. one FAPI-46-positive lesion, that could not be detected by SyngoVia Lesion Scout).

**Fig. 2:**
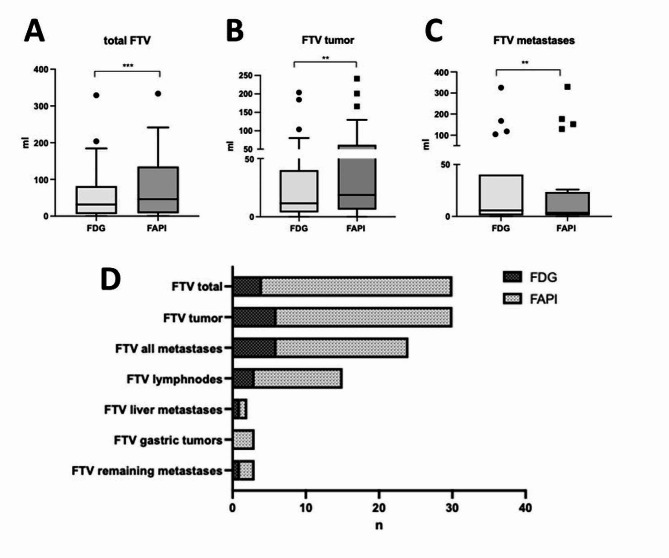
Functional tumor volumes (FTVs) Boxplot diagrams indicating functional tumor volumes (FTVs), **A**: total FTVs (primary tumors and metastasis), **B**: FTVs in primary tumors only, **C**: FTVs in metastasis only, **D**: Comparison of tumorous lesions (n), in which measured tumor volumes were larger in FDG (dark grey) vs. in FAPI-46 PET (light grey). FDG = fluorodeoxy-D-glucose, FAPI = fibroblast activation protein inhibitor

Median FTVs of suspicious lesions (primary tumor and metastatic lesions) were larger in FAPI-46-PET/CT (single or dual-tracer) than in FDG-PET/CT alone (*p* < 0.001). FTVs were 58.09 ± 76.20 ml and 77.04 ± 84.03 ml in FDG-PET/CT and FAPI-46 PET/CT (single and dual-tracer), respectively (see Table 3; Fig. 2).

**Table 3:** Functional tumor volumes (FTVs)

FTV	FAPI-46/dual-tracer PET/CT (ml, mean ± SD)	FDG-PET/CT (ml, mean ± SD)	Wilcoxon signed-rank test; Pearson’s correlation
FTV_total_	77.04 ± 84.03	58.09 ± 76.20	*p* < 0.001; *r* = 0. 0837
FTV_tumor_	49.17 ± 63.67	32.82 ± 49.71	*p* = 0.002; *r* = 0.703
FTV_metastases_	33.69 ± 74.64	40.03 ± 82.03	*p* = 0.008; *r* = 0.992

Functional tumor volume (FTV) of all tumorous lesions (FTV_total_), tumor (FTV_tumor_), and metastasis (FTV_metastases_) on FAPI-46/dual-tracer PET/CT and FDG-PET/CT for all patients. Volumes were compared using the Wilcoxon signed-rank test (Pearson’s correlation). FDG = fluorodeoxy-D-glucose, FAPI = fibroblast activation protein inhibitor

In primary tumors, FTV in FDG-PET/CT was 32.82 ± 49.71 ml vs. 49.17 ± 63.67 ml on FAPI-46 PET/CT (single and dual-tracer) (*p* = 0.002) (see Table 3; Fig. 2). Contrary to this, in metastasis, FTV was 40.03 ± 82.03 ml on FDG-PET/CT vs. 33.69 ± 74.64 ml in FAPI-46 PET/CT (smaller in FAPI-46, *p* = 0.008).

### GTVs measured on FAPI-46/dual-tracer PET/CT vs. FDG PET/CT

**Fig. 3:**
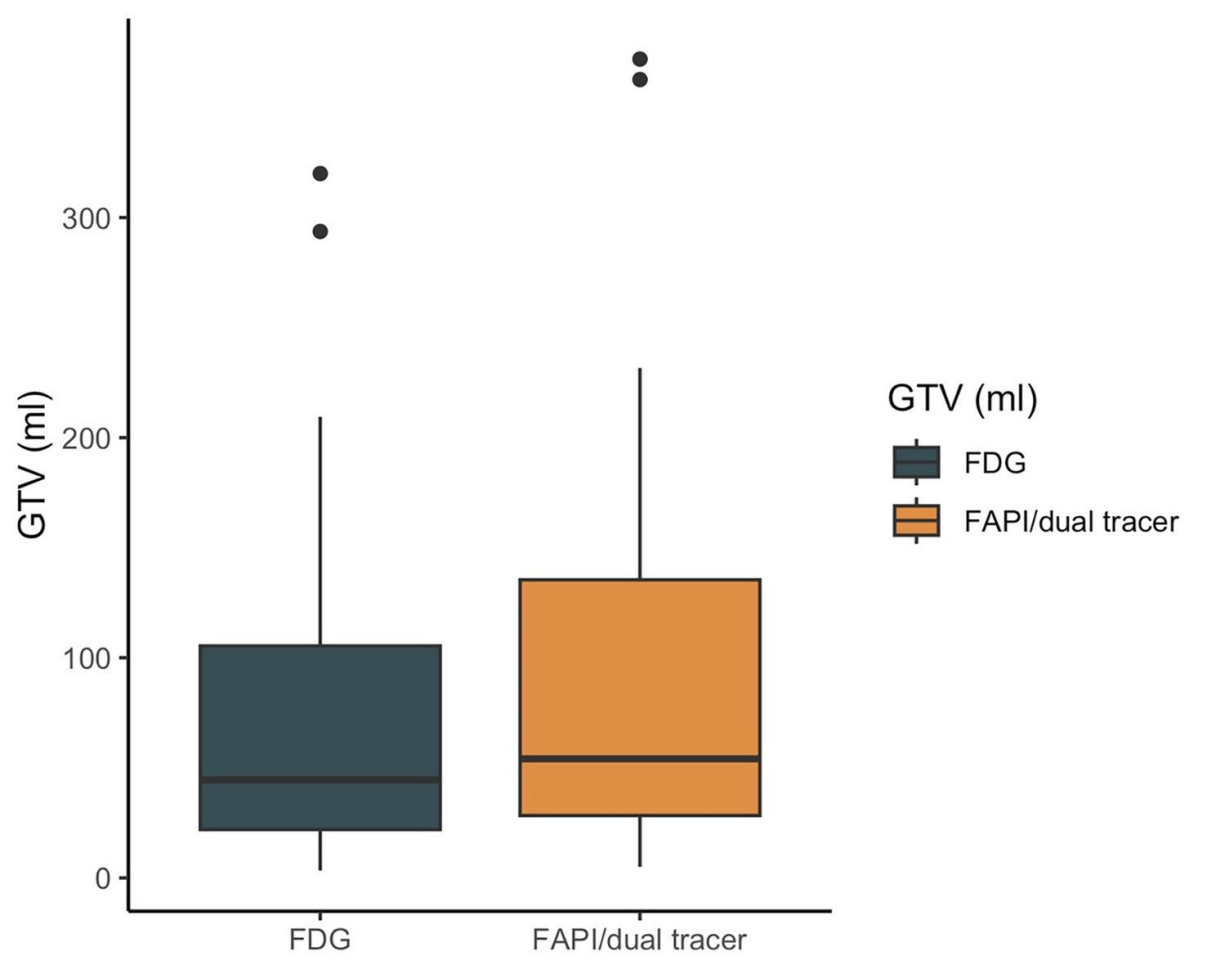
Gross Tumor Volumes (GTVs) Gross Tumor Volume (GTV) delineation (ml) according to FDG (green) and FAPI-46/dual-tracer PET/CT (yellow). All tumorous lesions (primary tumor, metastatic lymph nodes and, if present, distant metastasis with increased tracer accumulation and CT correlate) were contoured as GTV.

Target volumes were defined using VARIAN Eclipse software. Patients in this cohort were treated with neoadjuvant or definitive chemoradiation for esophageal cancer. We performed GTV measurements in all patients with FDG and FAPI-46 single- and dual-tracer PET/CT. All tumorous lesions (primary tumor, metastatic lymph nodes and, if present, distant metastasis with increased tracer accumulation and CT correlate) were contoured as GTV. In two patients, contouring GTV on basis of the FDG PET/CT was impaired by low FDG-accumulation compared to background tissue. Even when acknowledging endoscopic examinations and contrast-enhanced CT, insecurities about GTV delineation remained and local spread could preferably be determined in FAPI-46/dual-tracer PET/CT. GTVs were significantly larger in FAPI-46/dual-tracer scans compared to FDG PET/CT (mean 99.0 ml, SD 98.3 vs. mean 80.3 ml, SD 84.4, respectively (*p* < 0.001), see Fig. 3).

### TNM-Staging and change of treatment

Primary tumor was detected in all FAPI-46/dual-tracer scans and in 30/32 (93%) of FDG scans. Compared to the initial staging CT scan, 12/32 patients (38%) were upstaged in nodal status after the combination of FDG and FAPI-46 PET scans. 10/12 lesions were equally visible in both modalities (FDG and FAPI-46/dual-tracer-PET). FAPI-46/dual- tracer PET/CT led to changes in N-stage in 12/32 patients. Three metastatic lymph nodes were visible in FAPI-46/dual-tracer only. PET scans revealed new distant metastasis in 2/32 (6%) patients in the PET with FAPI-46: one patient with suspicion of liver metastasis and one patient with suspicion of a soft tissue metastasis in the right iliopsoas muscle. We decided to perform liver MRI to confirm metastasis and a biopsy from the metastatic region in the other patient’s iliopsoas muscle. Unfortunately, patient showed to have metastasis of esophageal cancer (adenocarcinoma), so, in both cases, we changed treatment regimen to palliative chemotherapy.

**Fig. 4:**
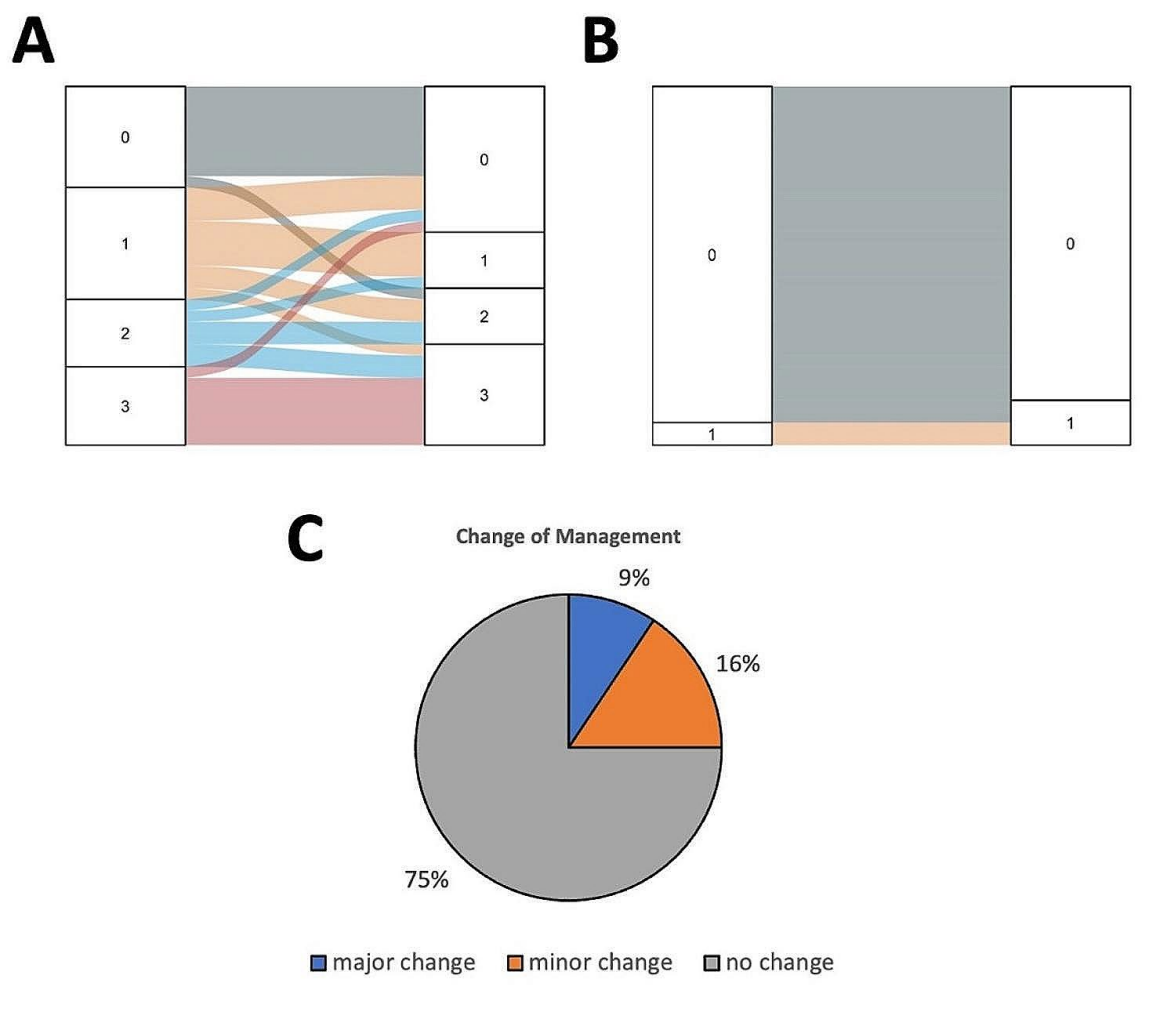
Changes in N-/M-staging after FAPI-46/dual-tracer PET/CT and changes of treatment Changes in N-/M-staging and changes of treatment, **A**: nodal status, box on the left: N-stage (0–3) according to initial CT staging, box on the right: N-stages (0–3) after FAPI-46/dual-tracer PET/CT; colored lines indicate stage migration **B**: M-stage, left box: M-stage according to CT-staging, right box: M-stage according to FAPI-46/dual-tracer PET/CT; **C**: changes of treatment, *“major”*: change of treatment regimen (e.g. from curate to palliative), *“minor”*: changes in RT-field (enlargement/inclusion of nodal areas after FAPI-46/dual-tracer PET/CT). FAPI = Fibroblast activation protein inhibitor

Our findings led to larger RT fields (“minor change”) in 5/32 patients (16%) and changed treatment regimen (“major change”) in 3/32 patients (9%). Figure 4 gives an overview on the changes of cN/cM-stages according to the PET modality and its impact on treatment (changes of management). Minor treatment changes consisted of enlargement or adaption of the RT field, reasons for major changes was newly diagnosed M1-stiuation (liver and soft tissue metastasis, see above, and one patient with widespread nodal metastasis that was considered unsuitable for surgery or definitive chemoradiation). These patients were treated with palliative chemotherapy.

### Histopathology

**Fig. 5:**
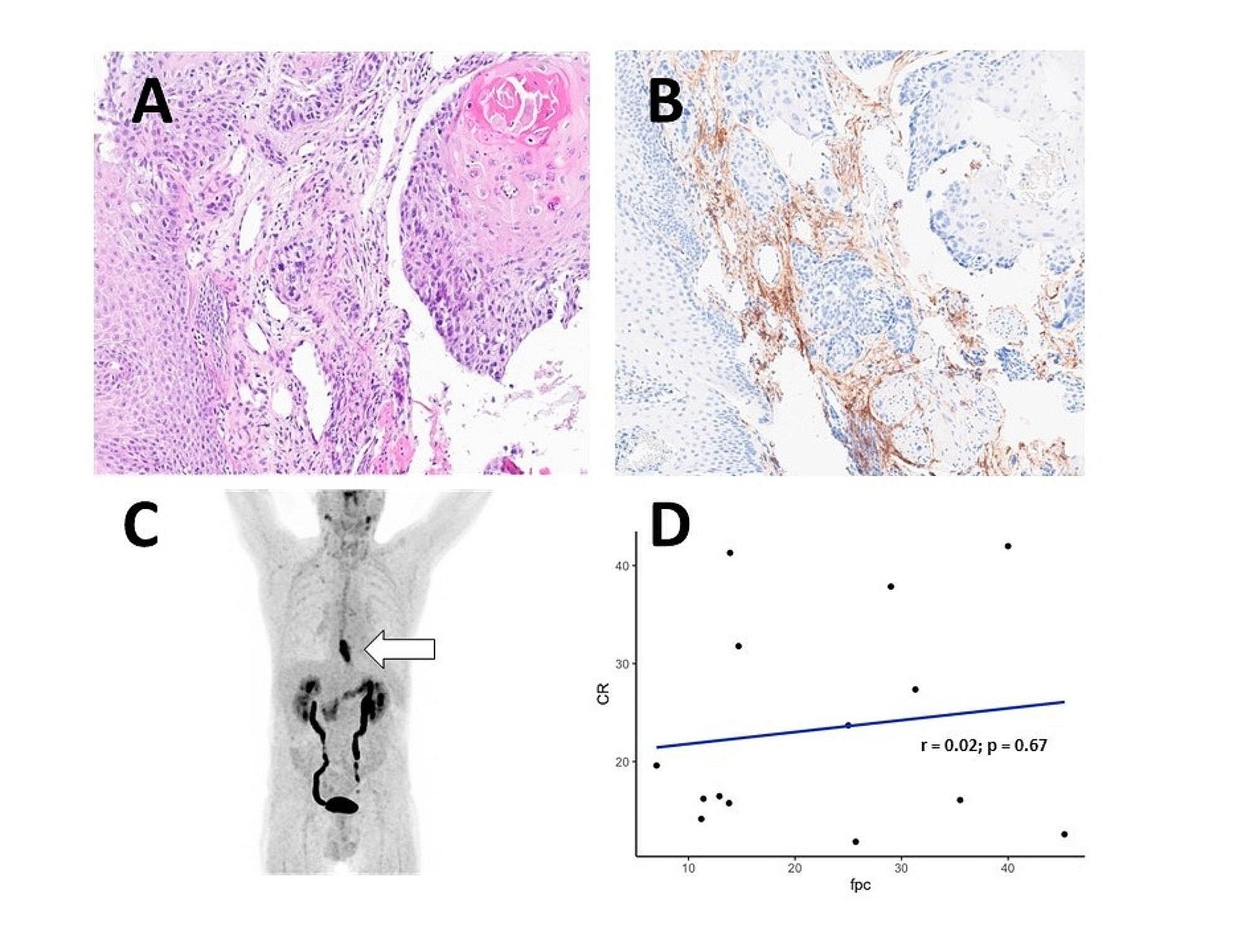
Tumor IHC staining and correlation between FAP-positive in cells (IHC) and FAPI-46/dual-tracer uptake (PET/CT) Patient with distal esophageal cancer, **A**: HE-staining of primary tumor, **B**: positive FAP-IHC of the same specimen, **C**: Maximum intensity projection (MIP) with FAPI-46/dual-tracer PET-positive tumor in projection on the distal esophagus, **D**: Correlation of CRmax (FAPI-46/dual-tracer PET/CT) and FAP-positive cells (“fpc”, in %) in the IHC

We accessed as many specimens as possible for secondary IHC (FAP staining) of the primary tumor tissue. We obtained 14/32 samples (43% of all patients) and observed heterogenous FAP-positivity in all specimen (between 7 and 45% of cells, IHC scoring (H-score) mean 36.3 (SD 24.6)). We correlated FAP positivity to the tumors’ CRmax rates but, in our small cohort, there was no correlation between high FAP-expression in the tissue and high FAP tracer uptake in the PET scans. An example of FAP-staining and correlation between FAP-positivity of cells (FPC) and CRmax rates is demonstrated in Fig. 5.

## Discussion

We report first data on the use of PET with FAPI-46 prior to radiotherapy. FAP has been shown to be overexpressed by cancer-associated fibroblasts (CAFs) in multiple epithelial cancers and other types of cancer and has been described for esophageal cancer before. Current literature is focused on FAP expression in epithelial cancers and there is limited data on FAP expression in adenocarcinoma or other histologies. In our cohort, more than half (53%) of patients had adenocarcinoma, with all primary tumors being FAP-positive. Of the tumors with secondary FAP-staining, 4/14 (28,6%) were adenocarcinomas and 71,5% (10/14) SCCs. Probably due to the small sample size, we were not able to detect a trend towards higher or lower FAP-expression in one or the other histological subtype. Prior studies, including a meta-analysis which evaluated multiple cancer types, found that high expression of FAP may be associated with poor outcomes [12]. According to the authors, this applies particularly, if FAP overexpression is found in the tumor cells rather than the stroma. FAP IHC is simple and inexpensive to use, so in the future it may be possible to stratify patients wo will benefit from FAP-diagnostics or FAP-treatment based on the IHC of the tumor biopsy. Although in our analysis we could not see a correlation between histopathology and PET scan, other groups were able to describe a correlation between positive FAP-staining in IHC and stronger FAP expression in the PET scan [13]. There is preliminary data supporting FAPI-Radioligand therapy (radiotheranostic approach) which has been applied in different tumor entities, mainly in sarcoma, but also in pancreatic cancer [14, 15]. 

Clinical studies comparing imaging with FDG versus FAPI PET/CT for various types of cancer are emerging and hint at a superior diagnostic efficacy of FAPI in diagnosing primary and metastatic lesions in patients [16]. However, these results should be regarded as preliminary and high-level evidence is still lacking. At present, special caution is required when upstaging a patient based on FAPI-imaging alone since there are known to be various benign causes of uptake (like benign tumors; fibrotic, degenerative or inflammatory diseases) [17–19]. At present, FAPI radiopharmaceuticals lack approval and their access may therefore be restricted to few clinical centers.

Recent data on external beam dose escalation (“boost”) in EC showed that dose application higher than 50 Gy is not beneficial for patients as it is associated with higher mortality due to treatment related toxicity [20–22]. In other solid tumors, such as non-small cell lung cancer (NSCLC), PET-based RT planning became standard [23]. Contrary to that, in EC, large RT fields with extended clinical target volume (CTV) and planning target volume (PTV) margins are considered standard of care and radiation of non-metastatic lymph nodes occurs (e.g. non-affected LN in proximity to affected LNs), even if PET/CT was performed prior to radiotherapy. However, there is increasing evidence that elective lymph node irradiation, irrespective of the exact tumor entity, may impair the immune response, potentially leading to worse treatment response after RT, chemotherapy or immunotherapy [24, 25]. A future approach could be PET-guided dose escalation (omitting RT in PET-negative areas and reducing GTV-to-CTV and CTV-to-PTV margins, by doing so: saving dose in organs at risk and no elective nodal irradiation). In NSCLC, high RT doses at the base of the heart were shown to correlate with worse overall survival [26]. Due to similar anatomical location, the strategy of cardiac dose sparing may be similarly beneficial to patient receiving esophageal RT. On the other hand, EC may need larger irradiation fields due to diffuse intra-esophageal growth patterns and discontinuous LN-spread (e.g. skip lesions in the esophageal mucosa) [27]. In most cases, there is no orderly outflow of lymph from the primary esophageal tumor into a first LN (principle of “sentinel node”, as well-established in breast cancer), but multiple level LN affection as well as skip metastasis in mediastinal lymph nodes are described [28]. Here, “PET-only” RT planning involves a risk of a higher rate of nodal tumor recurrence or progression in (initially) PET-negative areas. There is demand for prospective studies on PET planning (FDG and FAPI/dual-tracer) for EC in the neoadjuvant and definite setting. At present, special caution is required when upstaging a patient based on FAPI-46-imaging alone, since, as with FDG-imaging, there are known to be various benign causes of uptake: benign FAPI uptake is reported in to degenerative and traumatic bone and joint lesions, arthritis, but also in inflammation, infection (e.g. reactive lymph nodes), fibrosis and scar tissues, representing potential pitfalls in cancer staging [29]. 

## Conclusion

We report first data on the use of PET with FAPI-46 for patients EC, who are scheduled to receive RT. Tumor uptake was high and not depending on FAP-expression in TME. Further, FAPI-46 PET had relevant impact on management in this setting (led to minor or major changes of treatment in one fourth (25%) of patients). Our data calls for prospective evaluation of FAPI-46 PET to improve clinical outcomes of EC. Still, false-positive FAPI-46 PET/CT findings should be considered.

## Data Availability

No datasets were generated or analysed during the current study.
